# Application of Different Imaging Methods in the Early Diagnosis of Primary Hepatic Carcinoma

**DOI:** 10.1155/2016/8763205

**Published:** 2015-12-24

**Authors:** Xin'ai Wu, Jianbo Li, Cheng Wang, Guojian Zhang, Na Zheng, Xuemei Wang

**Affiliations:** ^1^Inner Mongolia Medical University, Hohhot 010050, China; ^2^Department of Nuclear Medicine, Inner Mongolia Medical University Affiliated Hospital, Hohhot 010050, China

## Abstract

Primary hepatic carcinoma (PHC) is the one of the most common tumors and the common cause of cancer death in the world. Detecting PHC in its early stage by imaging methods may greatly increase survival rates of patients. Ultrasound, computed tomography, magnetic resonance imaging, and positron emission tomography/computed tomography are common imaging methods in the diagnosis of PHC. In this paper, the application of different imaging methods in diagnosing the primary hepatic carcinoma will be discussed.

## 1. Introduction

Primary hepatic carcinoma (PHC) is the common liver tumor, including hepatocellular carcinoma (HCC) and intrahepatic cholangiocellular carcinoma (ICC). The survival rate of patients with PHC has decreased over the last years [1]. The PHC is caused, in part, by the epidemic of hepatitis B and hepatitis C viral infections [2–5], which can lead to cirrhosis and PHC. Detecting PHC in its early stage by imaging methods may provide patients with more opportunities for curative treatment and high survivability. Ultrasound (US), computed tomography (CT), and magnetic resonance imaging (MRI) are common methods in screening the liver tumors, most of which can be detected by these methods, but some atypical tumors of which can be uneasily detected in time. PET/CT is a molecule imaging method which can specifically detect the atypical tumors in some cases. Different imaging methods in diagnosing the PHC will be summarized as follows. Figures data in this paper were from Imaging Department of Inner Mongolia Medical University Affiliated Hospital. Human studies have been approved by the Institutional Review Board of Inner Mongolia Medical University as well as the local ethics committee. Written consents have been obtained from patients.

## 2. Detection of Primary Hepatic Carcinoma by US

Internal echo on US is changing in the accretion of liver tumor, and internal echo usually develops from low or equal echo to high or mixed echo. Therefore, US can accurately reflect the echo changes of PHC and it is an important imaging method to screen the PHC.

**Figure 1:**
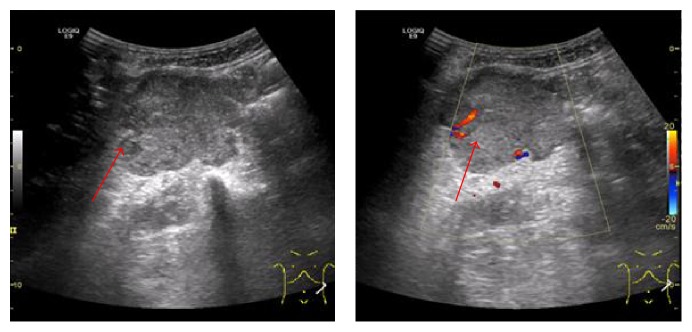
Ultrasound shows hypoechoic nodule in the hepatic lobe, and CDFI shows blood flow in the lesion. Lesion (red arrows) by liver puncture, which is guided by US, is proved to be PHC.

In the Doppler spectrum analysis, blood supply of liver tumor can be divided into hepatic artery, portal vein, and hepatic vein blood flow. The color Doppler flow imaging (CDFI) performance is peripheral or internal color blood flow signal of liver tumor (Figure 1). Although CDFI can detect the blood flow signal and the direction of blood flow and the distribution of the blood vessels, there are still insufficiencies, especially in the evaluation of deep tumors, tumors of slow blood flow, and few vessels.

Contrast enhanced US (CEUS) is widely used to diagnose liver tumor and it has important diagnostic value. Most of the PHC imaging modes on CEUS are the “fast forward” which is rapid arterial enhancement, and enhancement fades in portal or delay phase. In the study of Westwood et al. [6], the estimate of sensitivity and specificity for malignancies using CEUS was 95.1% and 93.8%, respectively. For the liver tumors, clinical CEUS [7] showed that the sensitivity and specificity were about 94.4% and 100%, respectively, and the accuracy increased from 54% to 96% after enhancement. Raza et al. [8] reported that sensitivity and specificity of CEUS in the detection of HCC with portal vein thrombosis were 95% and 83%, respectively. CEUS can significantly improve the detection rate of HCC.

In recent years, as US contrast agents and imaging technology are developing, CEUS can observe the tumor perfusion in real time, and dynamic enhanced performance can be analyzed after each phase of the enhancement, so as to provide the possibility for lesions qualitative analysis. CEUS has become a noninvasive method to assess liver tumor microcirculation and new vessel formation. However, in the study of Galassi et al. [9], there were much more cases in cirrhotic patients of misdiagnosing ICC for HCC in CEUS than that in CT (52% versus 4.2%) and that in MRI (52% versus 9.1%).

In conclusion, US and its related imaging technology (CEUS) have important clinical significance in the diagnosis of liver tumor, but it is difficult to identify the benign and malignant liver lesions and liver puncture guided by US should be taken further.

## 3. Detection of Primary Hepatic Carcinoma by CT

CT diagnosis of the PHC is mainly based on the configuration or size change, the density change, or signal difference between the lesion part and normal liver tissue.

**Figure 2:**
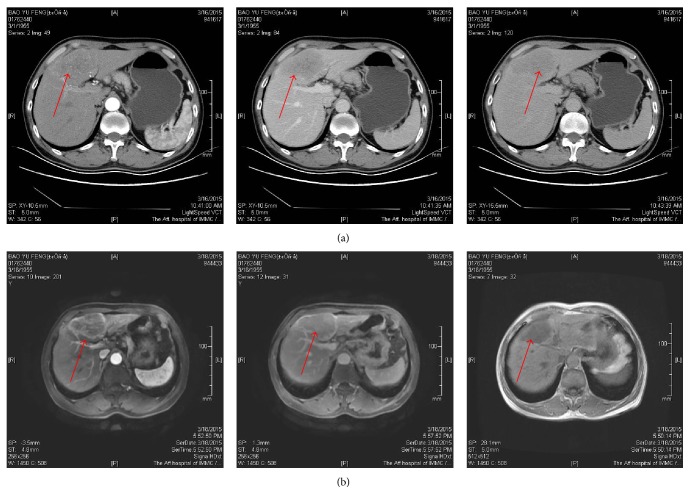
A 60-year-old man has a nodule of low density in the left hepatic lobe segment. (a) Multiphase CECT showed significant enhancement in arterial phase, the enhancement extent fade in portal and delay phase. (b) Multiphase contrast enhanced MRI showed typical enhancement pattern (red arrows in a, b). In conclusion, the lesion in the left hepatic lobe was consistent with PHC.

The applications of contrast enhanced CT (CECT) have greatly improved the diagnostic accuracy of the PHC. Not only can it show vascular perfusion status but also it can identify the benign and malignant lesions and its relationship with the surrounding blood vessels through CECT. The density difference between the lesion and normal liver tissue will be obvious after enhancement. The typical enhancement pattern of tumor is significantly strengthened in arterial phase and tumor enhancement disappears during the venous or equilibrium phase (Figure 2(a)). CECT has become the routine diagnosis method of PHC. Researches showed that the overall diagnostic sensitivity of CECT was 70%–74% [10, 11].

The study of Kanata et al. [12] found that CECT was a more suitable modality than enhanced MRI in evaluation of arterial blood supply in HCC. In the other study [13], the sensitivity, specificity, and accuracy of predicting poor differentiation in HCCs in arterial phase by CECT were 75%, 90%, and 88%, respectively, and in the venous phase these were 100%, 55%, and 60%, respectively. CECT is the most common used imaging method and has high accuracy in diagnosing typical HCC.

CT perfusion is a new method to analyze dynamic changes in liver tumors. Studies [14, 15] showed that hepatic blood flow, hepatic arterial perfusion, and hepatic portal perfusion were significantly increased in the tumor edges of HCC patients. CT perfusion of tumors may be helpful in revealing histopathological features, as well as indirectly reflecting angiogenic changes.

Because of the lesion size, blood supply, growing pattern, background, and composition of PHC, its manifestations in CECT are not typical in some cases. For atypical CT manifestations of the liver tumors, joint diagnosis should be taken in combination with other advanced imaging methods.

## 4. Detection of Primary Hepatic Carcinoma by MRI

MRI reflects physiological function information through the water molecules activities. MRI has high accuracy in liver tumor diagnosis, and it is a valuable diagnostic method in liver examination [16].

There are multiple sequences in MRI scan which can detect nodular lesion in the liver and accurately identify hemangioma and even small hepatocellular carcinoma. Small hepatocellular carcinoma, also known as early HCC, is defined as the maximum diameter of the single tumor nodule (less than 3 cm) or the sum diameters of two nodules (less than 3 cm). MRI has been widely used for early diagnosis and prognosis evaluation of small hepatocellular carcinoma. The research of Clasen et al. [17] pointed that detection rate of MRI and CT for small liver cancer (less than 3 cm in diameter) was 96.3% and 79.3%, respectively. Researches [18–20] reported that the sensitivity of MRI and CT in diagnosis of small hepatocellular carcinoma was 82.4%–89.8% and 57.6%–62.7%, respectively, and other researches [21, 22] reported that the sensitivity of MRI, CT, and CEUS were 64.1%–79%, 58%, and 45%–56.4%, respectively. These results from statistics showed that MRI could improve the detection rate of small hepatocellular carcinoma comparing with other imaging methods.

Diffusion weighted images (DWI) and enhanced MRI [23] can provide more valuable diagnosis information of liver tumors. DWI combined with DWI-conventional images could improve the diagnostic accuracy from 76.17% to 82.56% in diagnosis of HCC [24, 25].

After injection of gadolinium diethylenetriamine pentaacetic (Gd-DTPA) [26], MRI scanning could clearly show the blood perfusion of tumors. Golfieri et al. [27] found that the newly introduced MRI contrast agent Gd-DTPA had enabled the signal enhancement of tumor vascular during the hepatobiliary phase (HBP) (Figure 2(b)), and it could help to detect and characterize small HCCs. HBP-MRI identified hypovascular HCC nodules that were difficult to detect in US or CT. Some researches showed that the sensitivity and specificity of enhanced MRI in diagnosis of HCC were 78%–79.8% and 92%–96.8% [28–31]. Enhanced MRI has rapidly become a key imaging tool for the diagnosis of HCC.

However, MRI image quality could be affected by breathing, cardiopulmonary dysfunction, and elderly patients in poor condition. In addition, because of being affected by heatstroke and aortic artifacts, the lesions at the top of the diaphragm and the left hepatic lobe are hard to find. MRI related methods and MRI targeted contrast agents remain to be further researched in the PHC diagnosis.

## 5. Detection of Primary Hepatic Carcinoma by PET/CT

Currently, the diagnosis of PHC is mainly based on US, CT, MRI, and other imaging methods. US is mainly used for screening and biopsying guidance. Most PHC diagnosis mainly depends on CT and MRI. CT and MRI have certain advantages in judging tumor location, size, number, the internal structure, the vascular invasion, and lymph node metastasis, but it is often difficult to differentiate single benign and malignant nodule, and there are also limitations in these imaging methods, for example, evaluation of tumor differentiation degree and sensitivity of distant metastases detection. In addition, the early treatment plan of patients would be affected.

PET/CT imaging, as a kind of metabolic imaging method, mainly reflects the pathological changes, physiological or biochemical changes, and metabolic abnormalities in early stage of PHC. PET/CT imaging, combining functional and anatomic information, has been widely used in early diagnosis, staging, and evaluation of treatment, prognosis of the tumors.

^18^F-Fluorodeoxyglucose (^18^F-FDG) is the most widely used agent in PET/CT imaging, while there is still deficiency [32, 33] in diagnosis. Glucose metabolism presents normal in better differentiated HCC lesions and ^18^F-FDG imaging can hardly find lesions in these cases. Study [34] confirmed that ^18^F-FDG PET/CT imaging had high sensitivity in the diagnosis of bile duct carcinoma, but low sensitivity in HCC, only about 50%–60%. HCC frequently occurs in the patients who had chronic liver diseases, and ^18^F-FDG distributions are asymmetry in these lesions, which often affect the detection of the tumors. Therefore, how to make use of PET/CT to diagnose the HCC early is becoming a key clinical problem.

Early dynamic ^18^F-FDG PET/CT can diagnose HCC nodules with hypervascularization when other morphologic imaging modalities are unsuitable. In the study of Schierz et al. [35], patients with hypervascularization on CECT underwent liver early dynamic ^18^F-FDG PET/CT. SUVmax of tumor peak exceeded liver levels in 85% lesions.

^18^F-FDG PET/CT imaging in diagnosing HCC is mainly based on the glucose metabolism. In order to obtain high accuracy of HCC diagnosis, other metabolism patterns of tumor cells (such as protein and fatty acids) were introduced. Studies [36, 37] suggested that ^11^C-acetate (^11^C-ACE) could enter into tumor cells, and the amount was in positive correlation with the phospholipids membrane and fat synthesis, which could be used in the diagnosis of HCC. ^11^C-ACE PET/CT imaging can reflect tumor metabolism and is not affected by glucose phosphorylation, so it can be used for the negative ^18^F-FDG imaging of high differentiation, low grade malignant tumor imaging, making up for the inadequacy of ^18^F-FDG imaging as well as greatly improving the clinical diagnostic accuracy of HCC.

In the study of Cheung et al. [38], patients with HCC underwent both preoperative dual-tracers of ^18^F-FDG and ^11^C-ACE PET/CT imaging and CECT and then underwent liver transplantation. The results after surgery verified that the sensitivity and specificity in CECT were 43.8% and 66.7%, respectively, and the sensitivity and specificity of dual-tracers of PET/CT imaging were 93.8% and 100%, respectively. This research showed that ^18^F-FDG combining with ^11^C-ACE PET/CT imaging has good clinical application prospect in diagnosis of PHC.

**Figure 3:**
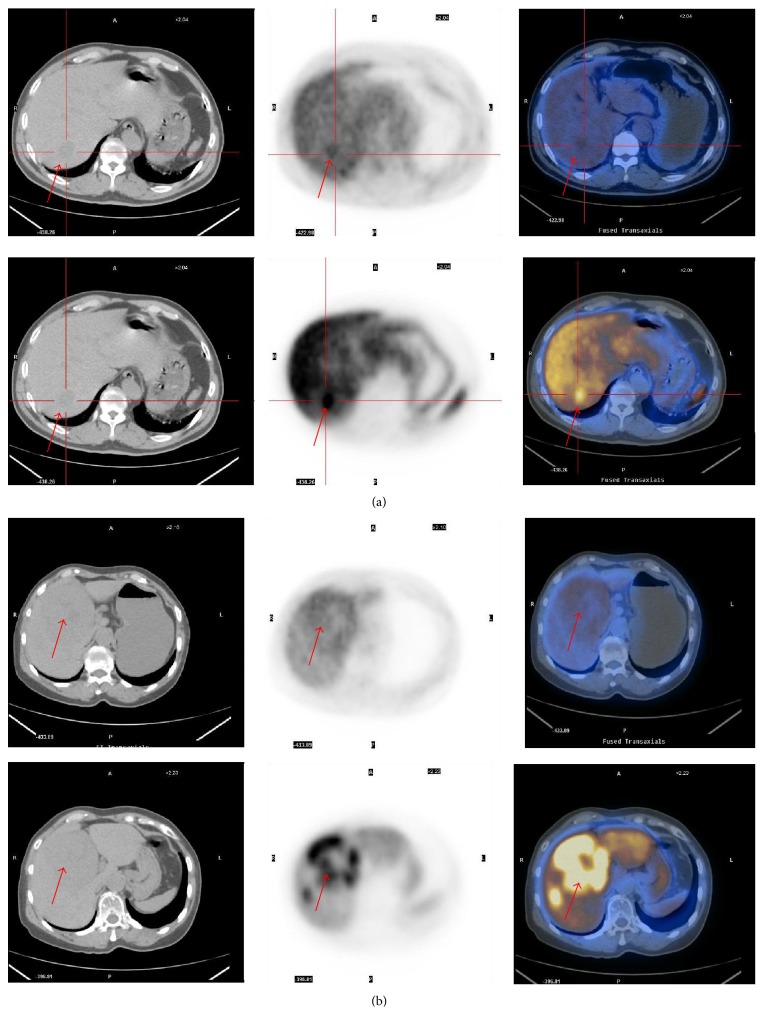
(a) ^18^F-FDG PET/CT images showed that lesion in the right hepatic lobe was ^18^F-FDG positive (SUV_max_: 2.4), and ^11^C-CHO PET/CT images showed that the uptake of lesion was increased (SUV_max_: 10.7) (red arrows on a). (b) ^18^F-FDG PET/CT image showed that lesion in the right hepatic lobe was ^18^F-FDG negative; ^11^C-CHO PET/CT image showed that the uptake of lesion was increased (SUV_max_: 12.8). These two cases in the right hepatic lobe were consistent with PHC (red arrows on b).

The study of Kornberg et al. [39] reported that the sensitivity of ^18^F-FDG PET/CT imaging in the diagnosis of HCC was low, and false negative results tended to appear in high grade differentiation of HCC. Studies [40–42] showed that ^11^C-choline (^11^C-CHO) could be used in diagnosis of high grade HCC. The research of Wu et al. [43] showed that the sensitivity in the combination of ^18^F-FDG and ^11^C-CHO PET/CT imaging was 89.0% in the diagnosis of HCC, compared with 63.1% in individual ^18^F-FDG PET/CT imaging. The research of Piert et al. [44] showed that ^11^C-CHO could be used for diagnosing a variety of malignant tumors, especially in prostate cancer and bladder cancer, and so forth. ^18^F-FDG in conjunction with ^11^C-CHO PET/CT imaging is significantly better than that of individual ^18^F-FDG PET/CT imaging in diagnosis of PHC (Figure 3).

## 6. Conclusions

Various imaging methods in the early diagnosis of PHC have advantages and disadvantages. US is a screening method of early PHC, and CT, MRI detection mainly reflects anatomic information. MRI and its related novel technologies are of high accuracy in diagnosis of small hepatocellular carcinoma. PET/CT imaging can provide functional and anatomic information of PHC. Researches in this paper showed that combining [45] PET/CT imaging, US, CT, and MRI could greatly improve the detection rate of PHC; however, there were still deficiencies in the diagnosis of early PHC. In conclusion, various imaging methods in the early diagnosis of PHC remain to be further researched, so as to prolong the long-term survival rate of patients.
